# Neutralizing Anti-IL-17A Antibody Demonstrates Preclinical Activity Enhanced by Vinblastine in Langerhans Cell Histiocytosis

**DOI:** 10.3389/fonc.2021.780191

**Published:** 2022-01-21

**Authors:** Selma Olsson Åkefeldt, Mohamad Bachar Ismail, Alexandre Belot, Giulia Salvatore, Nathalie Bissay, Désirée Gavhed, Maurizio Aricò, Jan-Inge Henter, Hélène Valentin, Christine Delprat

**Affiliations:** ^1^ Childhood Cancer Research Unit, Department of Women’s and Children’s Health, Karolinska Institutet, Stockholm, Sweden; ^2^ Astrid Lindgren Children’s Hospital, Karolinska University Hospital, Stockholm, Sweden; ^3^ UnivLyon, Université Claude Bernard Lyon 1, Villeurbanne, France; ^4^ Laboratoire Microbiologie Santé et Environnement, Doctoral School of Sciences and Technology, Faculty of Public Health, Lebanese University, Tripoli, Lebanon; ^5^ Faculty of Science, Lebanese University, Tripoli, Lebanon; ^6^ Centre International de Recherche en Infectiologie (CIRI), Univ Lyon, Inserm, U1111, Université Claude Bernard, Lyon 1, CNRS, UMR5308, ENS de Lyon, Lyon, France; ^7^ Pediatric Nephrology, Rheumatology, Dermatology Unit, HFME, Hospices Civils de Lyon, Bron, France; ^8^ Radiotherapy Unit, Department of Biomedical, Experimental and Clinical Sciences “Mario Serio”, University of Florence, Firenze, Italy; ^9^ Unité de recherche “Lymphoma Immuno-Biology”, Faculté de Médecine Lyon-Sud, Oullins, France; ^10^ Pediatrics, Azienda Sanitaria Locale, Pescara, Italy; ^11^ Centre de Recherche en Cancérologie de Lyon (CRCL) - INSERM U1052 - CNRS UMR5286 - Centre Léon Bérard, Lyon, France

**Keywords:** langerhans cell histiocytosis (LCH), survival, dendritic cells, cytokine, interleukin-17A (IL-17A), biotherapy and chemotherapy, vinblastine

## Abstract

Langerhans cell histiocytosis (LCH) is an inflammatory myeloid neoplasm characterised by the accumulation into granulomas of apoptosis-resistant pathological dendritic cells (LCH-DCs). LCH outcome ranges from self-resolving to fatal. Having previously shown that, (*i*) monocyte-derived DCs (Mo-DCs) from LCH patients differentiate into abnormal and pro-inflammatory IL-17A-producing DCs, and (*ii*) recombinant IL-17A induces survival and chemoresistance of healthy Mo-DCs, we investigated the link between IL-17A and resistance to apoptosis of LCH-DCs. In LCH granulomas, we uncovered the strong expression of BCL2A1 (alias BFL1), an anti-apoptotic BCL2 family member. *In vitro*, intracellular IL-17A expression was correlated with BCL2A1 expression and survival of Mo-DCs from LCH patients. Based on the chemotherapeutic drugs routinely used as first or second line LCH therapy, we treated these cells with vinblastine, or cytarabine and cladribine. Our preclinical results indicate that high doses of these drugs decreased the expression of Mcl-1, the main anti-apoptotic BCL2 family member for myeloid cells, and killed Mo-DCs from LCH patients *ex vivo*, without affecting BCL2A1 expression. Conversely, neutralizing anti-IL-17A antibodies decreased BCL2A1 expression, the downregulation of which lowered the survival rate of Mo-DCs from LCH patients. Interestingly, the *in vitro* combination of low-dose vinblastine with neutralizing anti-IL-17A antibodies killed Mo-DCs from LCH patients. In conclusion, we show that BCL2A1 expression induced by IL-17A links the inflammatory environment to the unusual pro-survival gene activation in LCH-DCs. Finally, these preclinical data support that targeting both Mcl-1 and BCL2A1 with low-dose vinblastine and anti-IL-17A biotherapy may represent a synergistic combination for managing recurrent or severe forms of LCH.

## Introduction

Langerhans cell histiocytosis (LCH) is a rare hematologic disorder, included in the “L” (Langerhans) group of histiocytosis by the Histiocyte Society (1). LCH cells were initially considered to be derived from Langerhans cells (LCs), a specialised subset of epidermal dendritic cells (DCs), according to their membrane co-expression of CD1a and Langerin (2). However, this origin has since been challenged by several results showing that LCH cells arise from different myeloid DC precursors rather than LCs exclusively (2–7). In 2008, we originally proposed that monocytes may be a source of pathological DCs (LCH-DCs) because monocyte-derived DCs (Mo-DCs) from LCH patients are abnormal. Indeed, they spontaneously undergo cell fusion under the control of their own IL-17A secretion to form long-lived multinucleated myeloid giant cells (MGCs), as observed within LCH lesions (8). Tissue-destructive LCH-DCs and derived giant cells were shown to accumulate and form granulomas; their structure being well delineated by CD1a staining, which was consequently used as a diagnostic marker (9). A minority of other cell types are also present in LCH granulomas such as macrophages, T cells and eosinophils. LCH granulomas can be found in all organs of the body, in particular in bone, with resorption areas observed in 80% of patients (1).

LCH is now classified as an inflammatory myeloid neoplasm, acknowledging the role of mutations in mitogen-activated protein kinase (MAPK) signalling pathways at different levels of myeloid precursor cells in fostering the disease. Interestingly, proliferating cells in LCH granulomas are mostly endothelial cells, fibroblasts, and T cells (10, 11), as CD1a^+^ LCH-DCs do not proliferate, albeit they display an extended lifespan due to the activation of survival pathways and inhibition of apoptosis (12, 13). This suggests that LCH-DCs accumulate *in vivo* to form granulomas and evolve into tumours following the aberrant long-term survival of LCH-DCs (11). Extended survival may result from the combination of intrinsic mutations in the MAPK signalling cascade of LCH-DCs (2, 14–16) with stimulations by abundant local and systemic inflammatory cytokines (17–24). The abnormal activation of the MAPK pathway has proven to be a deleterious mechanism in LCH, with 40-60% of patients harbouring the somatic *BRAFV600E* mutation (3, 5, 11, 25). Moreover, other rare mutations in *MAP2K1*, *ARAF* and *ERBB3* genes were detected (14, 26–28). Among actors of the cytokine storm, varying levels of interleukin-17A (IL-17A) were reported in LCH patient plasma (8, 21, 29–33), likely arising from circulating monocytes, as well as LCH-DCs and their derived giant cells inside granulomas (8, 29, 34, 35). IL-17A is assumed to be an important player in LCH pathogenesis as it induces Mo-DC fusion (8), and its plasma level is associated with LCH disease and sequelae (33). Interestingly, recombinant human IL-17A (rhIL-17A) induces *in vitro* transcription of BCL2-related protein A1 (BCL2A1, alias BFL1), an anti-apoptotic member of the BCL2 family, downstream of NF-κB activation in Mo-DCs from healthy donors (35, 36). Proteins of the BCL2 family regulate survival and sensitivity to apoptosis by modulating mitochondrial outer membrane permeabilization (37, 38). Accordingly, in a large number of hematopoietic cancer cells, *de novo* expression of pro-survival BCL2 proteins induces chemoresistance (37, 39, 40). Mcl-1 is the constitutive pro-survival factor of myeloid cells, including Mo-DCs (36). The pro-survival BCL2A1 member is highly regulated by the NF-κB pathway (39, 40). We have previously shown that recombinant human IL-17A (rhIL-17A) induces BCL2A1 expression; and, importantly, that Mo-DCs from healthy donors expressing both constitutive Mcl-1 and induced BCL2A1 treated with rhIL-17A acquired long-term survival and chemoresistance to 11 chemotherapeutic agents (35).

Inducing cell death of tissue-aggressive LCH-DCs is difficult but may be achieved in most patients by chemotherapy regimens containing vinblastine (VBL) and corticosteroids or, in salvage settings, cladribine (2CdA) and cytarabine (AraC) (41–44). Disseminated forms of LCH in the liver, spleen and hematopoietic system, especially in patients under 5 years of age are life-threatening (44). Indeed, among these cases, a third of patients fail to respond to first-line chemotherapy, including VBL. Despite the improvement in survival provided by MAPK pathway inhibitors, most therapies are not curative and result in a high rate of relapse after treatment (45, 46). Thus, novel therapeutic approaches are warranted with the aim of improving survival, reducing morbidity linked with central nervous system (CNS) involvement, and lowing treatment-related toxicity generally associated with intensive rescue chemotherapy.

In this study, we first explored BCL2A1 expression in LCH lesions and in Mo-DCs from LCH patients. We then investigated the preclinical activity of anti-IL-17A biotherapy on BCL2A1 expression in Mo-DCs from LCH patients. Finally, we evaluated the toxic activity of either VBL or 2CdA and AraC, combined with anti-IL-17A biotherapy. This *in vitro* study supports that the combination of low-dose VBL with anti-IL-17A biotherapy is an appealing therapeutic approach for LCH patients.

## Materials and Methods

### LCH Patient Samples

Patients with biopsy-proven LCH were enrolled in this study by two major clinical centres with expertise in LCH: Karolinska University Hospital, Stockholm (SE) and AOU Meyer, Florence (IT). The study was in agreement with the local ethics committees upon approval by their Institutional review boards. Informed consents were obtained. The characteristics of the study population are described in **Table 1**. We obtained blood samples from 20 patients with LCH (12 males and 8 females) from Sweden (n = 9) and Italy (n = 11). We carried out immunohistological studies on LCH bone lesions from Swedish patients exclusively (n = 2, p11 and p12).

**Table 1:** Main features of the patients with LCH.

Case^a^	Sex/Age at diagnosis^b^	Organs involved during course of disease ^c^	LCH chemo-immunotherapy received^d^	Age at study	Disease activity at evaluation^e^	Disease activity class^f^	Ongoing LCH chemo-immunotherapy at samplings	Sequelae^g^	% of intracellular BCL2A1 in Mo-DCs^h^
1 (a→b)	M/6 yr	Bone*, ears*, pituitary*, skin, nd	Local (extirpation) + VBL + CST + 6-MP + MTX → 2CdA → 6-MP + MTX + CST	18 → 20 yr	AD, chronic	2 → 1	6-MP + MTX + CST → 6-MP + MTX	Panhypopituitarism, DI, GHD, CNS-ND	49.7
2 (a→b)	M/4 yr	Bone*, pituitary*, nd	Local (steroids)	13 → 15 yr	NAD, sequelae	0	None	DI, GHD, CNS-ND	29
3	M/14 m	Bone*, skin*, spleen*	VBL+CST → 2CdA +ARAC	24 m	NAD	0	None	None	37
4	M/10 m	Skin*, bone, pituitary	CST + VBL + 6-MP	3 yr	Reactivation (skin and bone)	3	VBL + CST pulses	DI	30
5	M/7 m	Bone*, skin	Untreated	7 m	Active, diagnosis	3	None	None	31
6 (a→b)	F/2 m	Skin*, spleen	Local (steroids) → VBL + CST + 6-MP + MTX	4 → 6 yr	AD, chronic	1	6-MP + MTX	None	34.4
7 (a→b→c)	F/5 m	Bone*, skin, spleen, liver, bone marrow, thymus, pituitary, nd	CST → VBL + MTX + 6-MP → Etanercept + 2CdA + IVIG → VBL + 6-MP + MTX + CST → 6-MP + CST → VBL	10 →11→11 yr	Progression (CNS) → AD, Chronic → AD, Chronic	2 → 2 → 2	VBL →None → None	DI, GHD, CNS-ND	64
8	F/14 yr	Bone*, mm, lungs, pituitary	VBL + CST	15 yr	AD, better	1	VBL + CST	DI	37.5
9	M/2.4 yr	Bone*, mm, lungs, pituitary	VBL + CST	2.6 yr	AD, better	1	VBL + CST	DI	14.9
10	F/2.5 yr	Bone*, central nervous system*	VBL + CST	3 yr	AD, better	1	VBL + CST	DI, CNS-ND	12.9
11	M/19 yr	Bone *	Untreated	19 yr	Active	2	None	Walking impairment	na
12	F/4 yr	Bone*	Untreated	5 yr	NAD	0	None	None	96
13	M/8 m	Skin*, lymph node*, liver*, ears*, spleen, bone marrow, intestines, bone	VBL+CST, MTX, VP-16 → 2CdA +ARAC → VBL+MTX+6-MP+CST → MTX+6-MP	5 yr	AD, better	2	6-MP + MTX	None	97
14	M/2.8 yr	Skin*	Untreated	2.8 yr	Active, diagnosis	2	None	None	62
15	F/9.5 yr	Skin*	Untreated	9.5 yr	Active, diagnosis	2	None	None	54.8
16	M/3 yr	Bone*	VBL + CST	3.6 yr	AD, better	1	VBL + CST	None	23.7
17	M/1.5 yr	Bone*	VBL + CST	3.2 yr	AD, better	1	VBL + CST	None	75.3
18	F/17 m	Skin*, lymph nodes*, bone*	CST	19 yr	AD, Chronic	1	None	Coxarthrosis	86
19	F/7.6 yr	Bone*	untreated	7.6yr	Active, diagnosis	2	None	None	96
20	M/2 yr	Bone*	VBL + CST	3 yr	AD, better	2	VBL + CST	None	95

a → Sampled two or three times, a, b and c.

b M, male; F, female; yr, year; m, month.

c * indicates organ involved at diagnosis. nd, CNS involvement with neurodegeneration evidenced by MRI; Mm, mucous membranes.

d VBL, vinblastine; CST, corticosteroids; 6-MP, 6-mercaptopurine; MTX, methotrexate; local, local corticosteroid injection; 2CdA, Cladribine; ARAC, Cytarabine; VP-16, etoposide; IVIG, intravenous immunoglobulin → Second (or further) line treatment.

e AD, active disease (persistence of signs and symptoms; no new lesions); Chronic, Chronic disease; NAD, no active disease, resolution of all clinical signs and symptoms; Progression, progressive disease (progression of signs and symptoms and/or appearance of new lesions.

f Disease activity classes: 0, resolution (no signs of active disease); 1, mild (regression of active disease or mild chronic disease; no hypoalbuminemia or ESR elevation); 2, moderate (moderately active disease; mild thrombocytosis, hypoalbuminemia, or ESR elevation); 3, marked (progressive disease or constant markedly active disease; marked hypoalbuminemia or ESR elevation).

g DI, Diabetes insipidus; GHD, Growth hormone deficiency; CNS-ND, symptomatic CNS neurodegeneration.

h Detection of intracellular BCL2A1 expression in Mo-DCs from LCH patients was performed after immunostaining and flow cytometry analyses; “na”, not applicable.

### Reagents and Antibodies

Recombinant human GM-CSF, IL-4 and IL-17A were purchased from PeproTech (Neuilly-sur-Seine, France). Antibodies for flow cytometry: CD14, CD1a, HLA-DR, CD83, CD86 and isotype controls were purchased from Becton Dickinson (Le Pont de Claix, France), anti-IL-17A clone 41802 from R&D Systems (Minneapolis, USA), anti-BCL2A1 (3401 anti-A1) from BioVision (San Francisco, USA) and anti-Mcl-1 (Y37) from Abcam (Cambridge, UK). For biological assays, anti-IL-17A eBio64CAP17 was used (San Diego, USA). Toxic compounds were kindly provided by the Karolinska University Hospital pharmacy and their characteristics are listed in **Table 2**.

**Table 2:** *In vitro* and *in vivo* characteristics of chemotoxic drugs used in this study.

Class	Abbreviation: name	Clinical dose(µM)^a^	*In vitro* dose used (µM); [range], low, high ^b^	Targets
Alkaloid	VBL: Vinblastine	1.5	[0.06 – 60], 0.06, 0.6	Microtubule function
Pyrimidin analogue	AraC: Cytarabine	14 - 140	[0.8 – 800], 4, 40	DNA synthesis, Mcl-1
Purine analogue	2CdA: Cladribine	0.02	[0.00035 – 3.5], 0.3, 3	DNA synthesis

a Calculation of physiological doses: the magnitude of the microenvironment concentration around cells, in vivo, following clinical dose administration, was calculated by approximating that the drug could be distributed in half of the body aqueous volume (30L) with the formula: [(injected concentration) x injected volume]/30. The results are in the range of those indicated by pharmacokinetics studies.

b High dose corresponds to optimal dose for killing in vitro IL-17A-stimulated Mo-DCs (35).

### Immunohistofluorescence Labeling

4-µm paraffin-embedded bone biopsies were deparaffinized and rehydrated. Following epitope retrieval, tissue sections were incubated for 30 minutes in phosphate buffered saline (PBS-1x) plus 1% bovine serum albumin (BSA) with 3% human serum to block Fc receptors. They were then incubated overnight at 37°C with primary antibodies in a humid chamber. Replacement of the primary antibodies by non-relevant antibodies of the same immunoglobulin isotype was used as negative control. We used the following primary antibodies: mouse anti-CD1a (Acris Antibodies, DM363, 1:20 dilution) and rabbit anti-A1 (BCL2A1, Biovision, 3401-100, 4 μg/mL). The isotype control antibodies were mouse IgG1 (Dako, X0931) and Rabbit IgG (R&D, AB-105-C). Slides were then washed three times in PBS-1x plus 1% BSA and detection of the primary antibodies was performed with suitable isotype-specific secondary Alexa Fluor 488 and 647-conjugated antibodies (10 μg/mL, Invitrogen) for 30 minutes. Following three washes in PBS-1x plus 1% BSA, sections were mounted using Mowiol and then analysed by confocal microscopy using a Carl Zeiss MicroImaging Inc. LSM 510 confocal microscope. Image acquisition was performed using MetaMorph 7.0 Software (Molecular Devices).

### Mo-DC Differentiation and Cultures

CD14^+^ monocytes were purified (>95% CD14^+^) from the peripheral blood of LCH patients by ficoll and percoll gradients, followed by negative magnetic depletion of cells expressing CD3 or CD56 or CD19. Monocytes were treated 6 days with 50 ng/mL GM-CSF and 500 U/mL IL-4 in RPMI (Life Technologies, Carlsbad, CA, USA) supplemented with 10% FCS, 10 mM Hepes, 2 mM L-glutamine, 40 µg/mL gentamicin (Life Technologies) (47). Cytokines were then removed by washing DCs twice in cytokine-free medium. Flow cytometry analysis was routinely used for quality control of the *in vitro* immature DC phenotype CD14^-^CD1a^+^MHC-II^+^CD83^-^ (>98%). On day 0, Mo-DCs were seeded at 4,800 cells/mm^2^ and cultured for 7 days. Neutralizing anti-IL-17A antibody (eBio64CAP17 from eBioscience) or isotype control (Becton Dickinson) used at 15 µg/mL were added at the beginning of Mo-DC culture (8). VBL and AraC + 2CdA were respectively added 24 and 48 hours before the apoptosis assay, performed on day 7. IL-17A, BCL2A1 and Mcl-1 expression were measured 12 hours before the apoptosis assay.

### Flow Cytometry Staining

Immunostaining of cells was performed in 1% BSA and 3% human serum in PBS-1x. We stained the Mo-DCs for their intracellular IL-17A expression, using two different neutralizing anti-IL-17A mouse monoclonal antibodies, previously validated in biological assay (8). We used 2 µg/mL of primary anti-IL-17A, anti-BCL2A1 and anti-Mcl-1 and secondary PE-F(ab’)_2_ goat to mouse IgG (115-086-062, Jackson Immunoresearch, West Grove, PA, USA) antibodies. For intracytoplasmic staining, we blocked the Golgi apparatus with BD GolgiStop™, fixed and permeabilized the cells with the Cytofix/Cytoperm reagents according to procedures from the manufacturer (Becton Dickinson). Fluorescence was quantified on a LSRII (Becton Dickinson) and analysed using FlowJo software.

### Apoptosis Assay by Flow Cytometry

Cell survival was analysed by flow cytometry after DiOC_6_ (3,39-diexyloxacarbocyanine, (Molecular Probes) and propidium iodide (PI, Sigma-Aldrich) double staining. Cells were incubated 15 minutes at 37°C with 40 nM DiOC_6_ in culture medium to evaluate mitochondrial transmembrane potential (Δψ*m*). Viable cells have stable Δψ*m* whereas Δψ*m* decreases with cell commitment to apoptosis. 0.5 µg/mL PI was added before flow cytometry analysis of the cells and incorporated into DNA of dead cells whose membrane is permeabilized. Apoptotic cells are $\text{DiO}{\text{C}}_{6}^{-}\text{P}{\text{I}}^{+},$ while living cells are $\text{DiO}{\text{C}}_{6}^{+}\text{P}{\text{I}}^{–};$ 10^6^ DCs/well (survival >98%) were seeded at 4,800 cells/mm^2^ at day 0. The total number of viable cells per well was quantified by a time-monitored flow cytometry analysis during 2 minutes at high speed (1 µL/s). Cell survival was calculated as the percentage of viable cells at day 7 related to day 0 for 10^6^ Mo-DCs introduced at day 0. In the absence of cell division, cell death percentage is the complement of the survival percentage to 100.

### Statistical Analysis

Linear statistical analyses were applied to detect correlation by using Excel software. Three to five groups were compared with the Kruskal-Wallis test with Steel-Dwass-Critchlow-Fligner post-test, and p values were calculated using the XLSTAT-Biomed module software (Version 19.6, Addinsoft).

## Results

### Improved Delineation of LCH Granulomas With BCL2A1 Rather Than CD1a Staining

**Figure 1:**
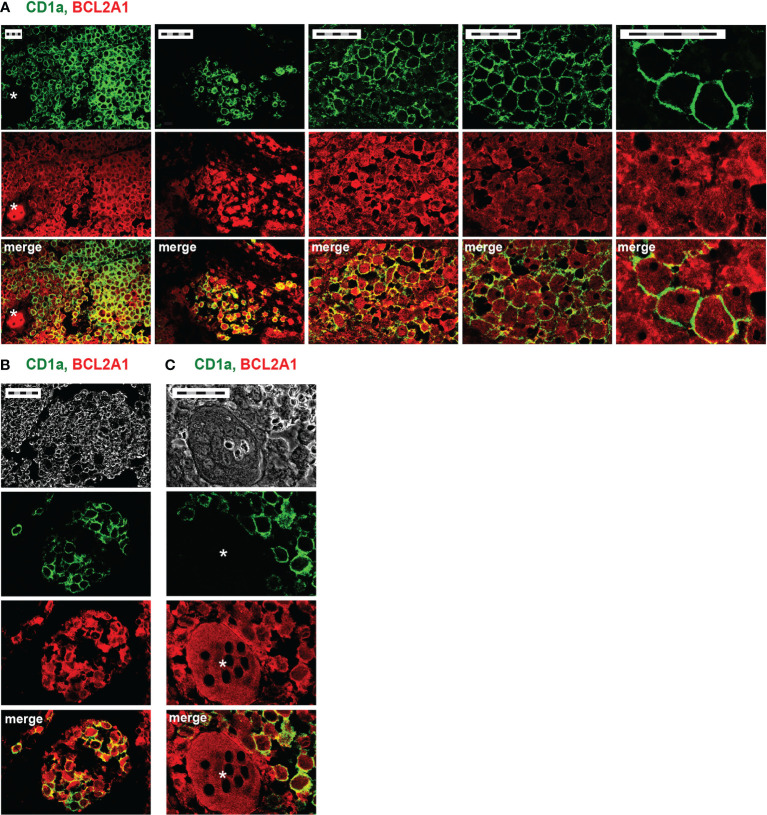
CD1a and BCL2A1 expression in LCH lesions after immunohistofluorescence staining. Representative confocal microscopy images of immunofluorescence staining on bone lesions from two patients with LCH (**A,** p12 and **B, C,** p11). Staining of CD1a (green, DC marker), BCL2A1 (red), and their co-localization (yellow) are shown. **(A)** Pathogenic LCH-DCs co-express CD1a and BCL2A1. Different magnifications of the same lesion focusing on mononucleated cells. **(B)** Improved delineation of LCH granulomas using BCL2A1 compared to CD1a. **(C)** Multinucleated giant cells (MGCs) expressing BCL2A1 in bone granuloma. *indicates MGCs. Scale bars: 50 µm (5 x 10 µm).

The diagnosis of LCH currently relies on the detection of CD1a expression. We previously documented that *(i)* IL-17A is expressed by DCs and MGCs inside LCH lesions (8) and *(ii)* rhIL-17A is able to induce BCL2A1 expression in Mo-DCs from healthy donors (35). We hypothesized that pathogenic DCs from LCH lesions may express both CD1a^+^ and BCL2A1^+^. To address this, we double stained CD1a and BCL2A1 in LCH lesions from two patients (p11, p12) (**Table 1** and **Figure 1**). Confocal microscopy analysis showed that CD1a was expressed at the membrane, whereas BCL2A1 was expressed in the cytoplasm of mononucleated LCH-DCs (**Figure 1A**). BCL2A1 was expressed in all CD1a^+^ LCH-DCs. Interestingly, BCL2A1 was also expressed in other CD1a^–^ cells in LCH samples, which may be CD1a^–^CD68^+^ and CD1a^–^CD14^+^ myeloid cells (48). Hence, LCH granulomas were better highlighted through BCL2A1 staining rather than CD1a expression in both mononucleated cells (**Figure 1B**) and in MGCs (**Figures 1A, C**, asterisk).

We provide evidence that pathogenic DCs and MGCs that accumulate in LCH lesions strongly express BCL2A1.

### Both IL-17A and BCL2A1 Expression Are Correlated With Survival of Mo-DCs From Patients With LCH

**Figure 2:**
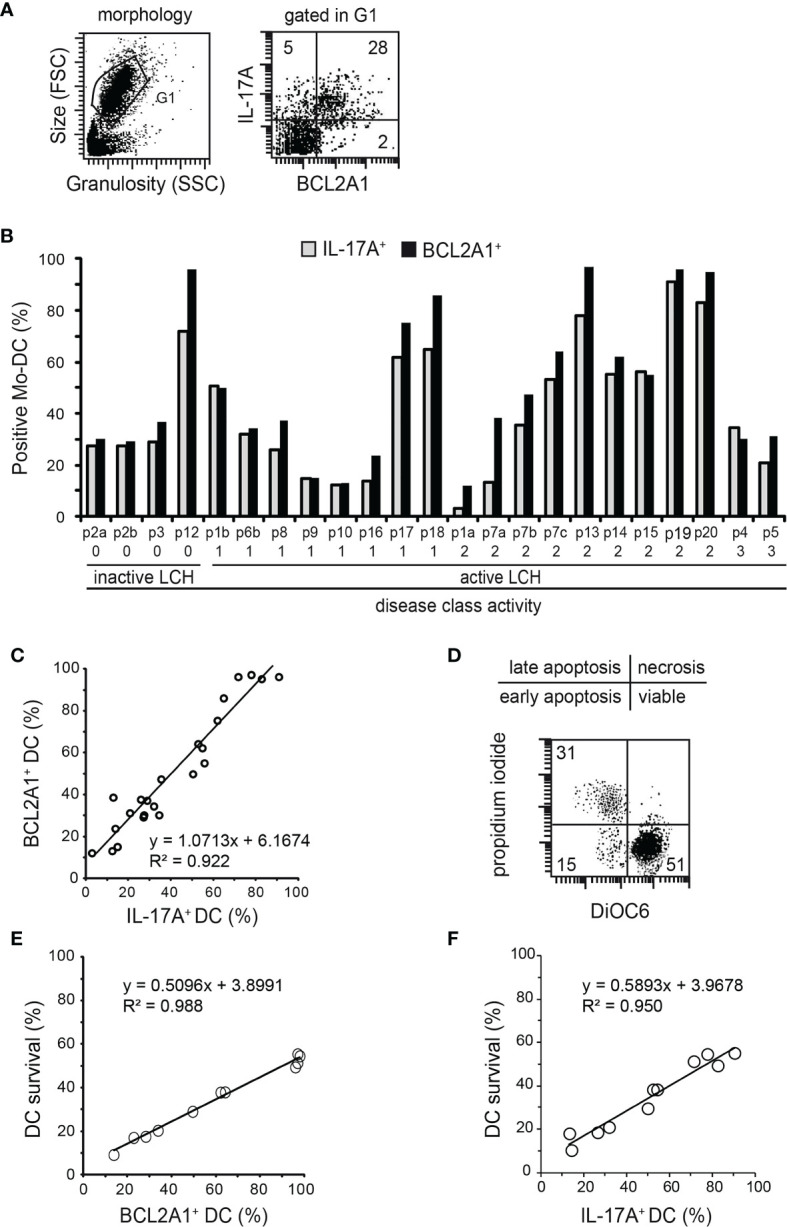
Statistical relationships between intracellular IL-17A and BCL2A1 expression, and/or Mo-DC survival from LCH patients. Flow cytometry analyses after intracellular staining of IL-17A and BCL2A1 in Mo-DCs from LCH patients (n=23 samples from 20 patients). **(A)** Representative dot plots of Mo-DC morphology (left) and their expression of BCL2A1 versus IL-17A (right). The numbers indicated in the dot plot correspond to the percentage of the positive cells. **(B)** Percentages of Mo-DCs expressing IL-17A and BCL2A1. Patients were plotted after ranking individuals according to their disease activity classes (as reported in **Table 1**): 0, Resolution; 1, Mild; 2, Moderate; 3, Marked disease. SD were below 2%. **(C)** Correlation between IL-17A and BCL2A1 expression in Mo-DCs from LCH patients. **(D)** Representative dot plot of DiOC_6_PI survival analyses. Percentage of viable Mo-DCs from LCH patients were quantified after 7 days in culture (initial density of one million Mo-DCs). $\text{DiO}{\text{C}}_{6}^{+}\text{P}{\text{I}}^{–}$ are viable Mo-DCs from LCH patients. **(E)** Correlation between BCL2A1 expression and survival of Mo-DCs from LCH patients. **(F)** Correlation between IL-17A expression and survival of Mo-DCs from LCH patients. **(C, E, F)** Linear regression statistical analyses were performed. y=f(x) indicates the equation of the statistical line of tendency and R^2^ the correlation factor.

Our previous studies indicated that blood myeloid cells from healthy donors did not express intracellular IL-17A or BCL2A1, while IL-17A was expressed in blood myeloid cells from LCH patients (8, 29, 34, 35). We herein analysed both IL-17A and BCL2A1 expression in Mo-DCs from the blood of LCH patients by flow cytometry (**Figures 2A, B**). Our findings substantiate previous studies, as we detected an intracellular expression of IL-17A (mean 45%, range 4-91%). A heterogeneous intracellular BCL2A1 protein expression was also detected in Mo-DCs from LCH patients (mean 53%, range 13-97%). BCL2A1 mRNA expression was detected both in IL-17A-treated Mo-DCs from healthy controls and in IL-17A-producing Mo-DCs from LCH patients, but not in untreated Mo-DCs from healthy controls (**Supplementary Figure 1**). These results suggest that BCL2A1 was not induced by GM-CSF plus IL-4 during Mo-DC differentiation. Interestingly, double staining performed on Mo-DCs from eleven patients demonstrated that IL-17A^+^ Mo-DCs from LCH patients co-expressed BCL2A1 (**Figure 2A** right and data not shown). Similarly to IL-17A expression [**Figure 2B** and (8)], BCL2A1 expression in Mo-DCs from LCH patients was correlated neither with the disease activity class nor with any chemotherapeutic treatment (**Table 1**). Strikingly, in Mo-DCs from each patient, the percentage of BCL2A1 expression paralleled the percentage of IL-17A expression (**Figure 2C**), suggesting that, as observed with Mo-DCs from healthy donors (35), BCL2A1 expression is under the control of IL-17A.

We previously documented that Mo-DCs from healthy donors cultured in medium spontaneously undergo apoptosis after two days of culture, with less than 5% of the Mo-DCs being viable at day seven (35). Here, we investigated whether BCL2A1 expression was correlated with Mo-DC survival in LCH patients, by measuring viable cells *via* $\text{DiO}{\text{C}}_{6}^{+}\text{P}{\text{I}}^{–}$ uptake by flow cytometry at day seven (**Figures 2D, E**). The percentage of viable Mo-DCs was linear to the percentage of BCL2A1-expressing Mo-DCs (**Figure 2E**, mean 33% and range 11-48% for survival). Moreover, viable Mo-DCs were also correlated with the percentage of IL-17A-expressing Mo-DCs in LCH patients (**Figure 2F**, mean 35% and range 10-55% for survival). Of note, we observed a heterogeneity in BCL2A1 and IL-17A expression among LCH patients (**Figures 2B–F**).

Taken together, our results indicate that Mo-DCs from LCH patients display an increased survival rate correlated with IL-17A and BCL2A1 expression, suggesting that BCL2A1 increases DC survival and participates in the accumulation of viable LCH-DCs within lesions.

### Routinely Used Chemotherapeutic Compounds Decrease *In Vitro* Survival of Mo-DCs From LCH Patients Independently of BCL2A1 Expression

**Figure 3:**
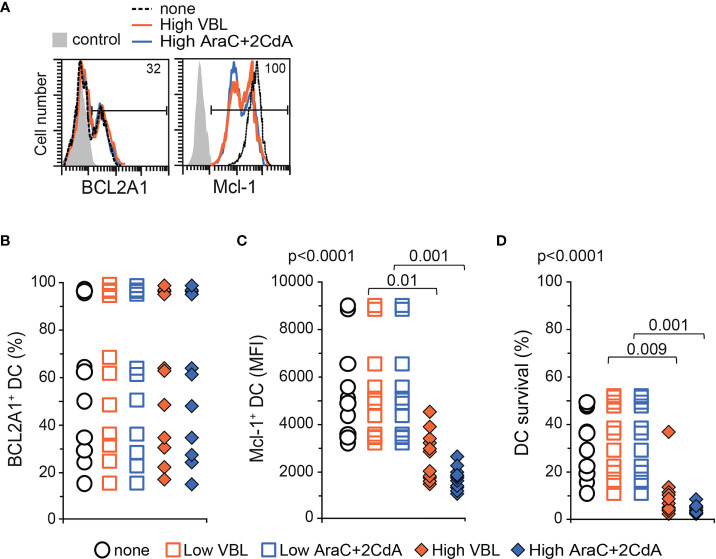
BCL2A1 expression and survival in Mo-DCs from LCH patients after treatment with chemotoxic compounds. High doses of drugs correspond to 0.6, 40 and 3 μM for VBL, AraC and 2CdA, respectively. Low doses are 10 times lower. BCL2-member expression and survival were measured by flow cytometry in Mo-DCs with LCH. **(A)** Representative flow cytometry analyses of BCL2A1 and Mcl-1 intracellular staining after incubation with either medium alone (none, dotted black line) or high doses of VBL (full orange line) or AraC and 2CdA (full blue line). Percentage of positive Mo-DCs from LCH patient is indicated inside each histogram. Control (grey) corresponds to isotopic control at staining. Percentages of intracellular **(B)** BCL2A1 and **(C)** Mcl-1 expression in the Mo-DCs from LCH patients after culture with either medium alone (none) or with low or high doses of VBL (Orange) or AraC and 2CdA (Blue). For Mcl-1 quantification, the mean fluorescence intensity (MFI) was more informative than the percentage because >95% of Mo-DCs expressed Mcl-1 in the absence of toxic compounds. **(D)** Percentage of $\text{DiO}{\text{C}}_{6}^{+}\text{P}{\text{I}}^{–}$ viable Mo-DCs from LCH patients treated or not with toxic drugs were quantified after 7 days of culture (initial density of one million Mo-DCs). **(B–D)** The mean of triplicate values was plotted for each patient. SD were below 2%. One symbol corresponds to one patient. Mo-DCs from 11 LCH patients (p1b, p2b, p7c, p6b, p9, p12, p13, p14, p16, p19, p20) were analysed. Statistical analyses: the Kruskal-Wallis test with Steel-Dwass-Critchlow-Fligner post-test were used to compare the groups and calculate the p values.

According to the standard-of-care, VBL is the main first-line treatment for disseminated LCH, and the combination of AraC and 2CdA is indicated as a second-line treatment (43, 44, 49). We investigated the ability of these agents (VBL alone or the combination of AraC and 2CdA) to decrease BCL2A1 expression and subsequently the survival of Mo-DCs from LCH patients by flow cytometry (**Figure 3** and **Table 2**). One or two days of exposure of Mo-DCs to low or high doses of these toxic compounds did not alter BCL2A1 expression (**Figures 3A, B** and **Supplementary Figure 2**). However, as previously shown for Mo-DCs from healthy controls (35), high doses of VBL decreased intracellular Mcl-1 expression in Mo-DCs from LCH patients (**Figures 3A, C**). Similar results were obtained with high doses of AraC and 2CdA. High doses of VBL impaired survival of Mo-DCs in 10 out of 11 LCH patients (**Figure 3D**). Similarly, high doses of AraC and 2CdA limited survival of Mo-DCs from all LCH patients (**Figure 3D**). Low doses of toxic drugs had no detectable effect on cell survival (**Figures 3C, D**).

Our preclinical results indicate that chemotherapeutic agents directed against LCH, when used at high doses, decreased Mcl-1 expression and killed Mo-DCs from patients, without affecting BCL2A1 expression.

### Neutralizing IL-17A Antibodies Inhibit BCL2A1 Expression and Impair Survival of Mo-DCs From LCH Patients

**Figure 4:**
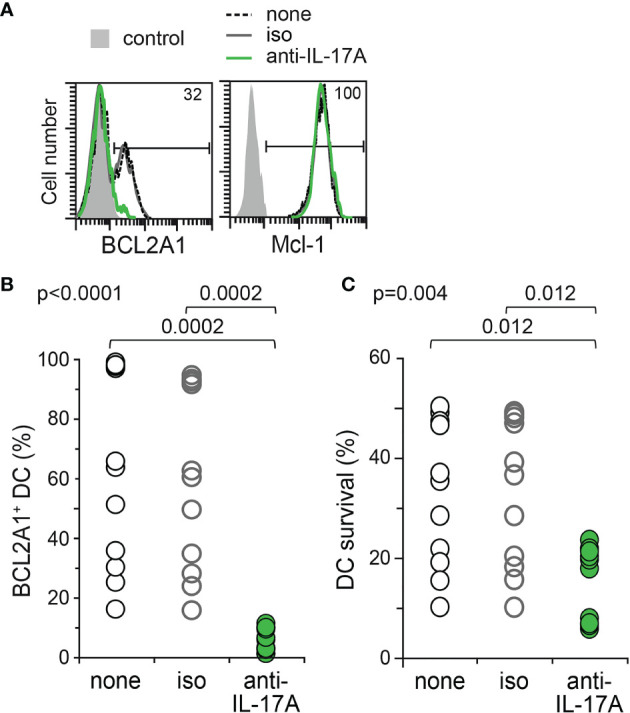
BCL2A1 expression and survival of Mo-DCs from LCH patients after neutralization of endogenous IL-17A. Mo-DCs from LCH patients were cultured 7 days in the presence of either medium alone (none, Black) or IgG1 isotype control (iso, Grey) or neutralizing anti-IL-17A Abs (anti-IL-17A, Green). **(A)** Representative flow cytometry analyses of BCL2A1 and Mcl-1 intracellular staining. Percentages of positive Mo-DCs from LCH patient are indicated. Control (filled grey histogram) corresponds to flow cytometry isotopic control. **(B)** Percentage of intracellular BCL2A1 expression in the Mo-DCs from all LCH patients tested. **(C)** Percentage of $\text{DiO}{\text{C}}_{6}^{+}\text{P}{\text{I}}^{–}$ viable Mo-DCs from the LCH patients per million cultured Mo-DCs. **(B, C)** The mean of triplicate values was plotted for each patient. SD were below 2%. One symbol corresponds to one patient. Mo-DCs from 11 LCH patients (p1b, p2b, p7c, p6b, p9, p12, p13, p14, p16, p19, p20) were analysed. Statistical analyses: the Kruskal-Wallis test with Steel-Dwass-Critchlow-Fligner post-test were used to compare the groups and calculate the p values.

BCL2A1 is known to confer chemoresistance, and is of poor prognosis in inflammatory disorders and haematological malignancies (36, 37, 39, 40). We previously showed that rhIL-17A induced BCL2A1 expression in healthy donor Mo-DCs (35). In order to study whether IL-17A confers a survival advantage to the Mo-DCs of LCH patients thanks to the BCL2A1 induction, we then cultured these Mo-DCs either in medium alone or in medium containing isotype control or neutralizing anti-IL-17A antibodies. BCL2A1 expression and survival of Mo-DCs from patients were then quantified by flow cytometry (**Figure 4**). Neutralizing anti-IL-17A antibodies, but not the isotype control, strongly inhibited the intracellular expression of BCL2A1 in Mo-DCs from LCH patients (**Figures 4A, B**). Interestingly, survival of Mo-DCs from LCH patients was drastically impaired by neutralizing IL-17A (**Figure 4C**). Consistently, survival was maintained in Mo-DCs from LCH patients with the highest IL-17A and BCL2A1 expression (data not shown).

Therefore, neutralizing anti-IL-17 antibodies impact BCL2A1 expression and Mo-DC survival in the context of LCH, indicating that the pro-inflammatory cytokine IL-17A is a major pro-survival signal for Mo-DCs from LCH patients.

### Neutralizing Anti-IL-17A Antibodies Increase *In Vitro* Chemosensitivity of Mo-DCs From LCH Patients

**Figure 5:**
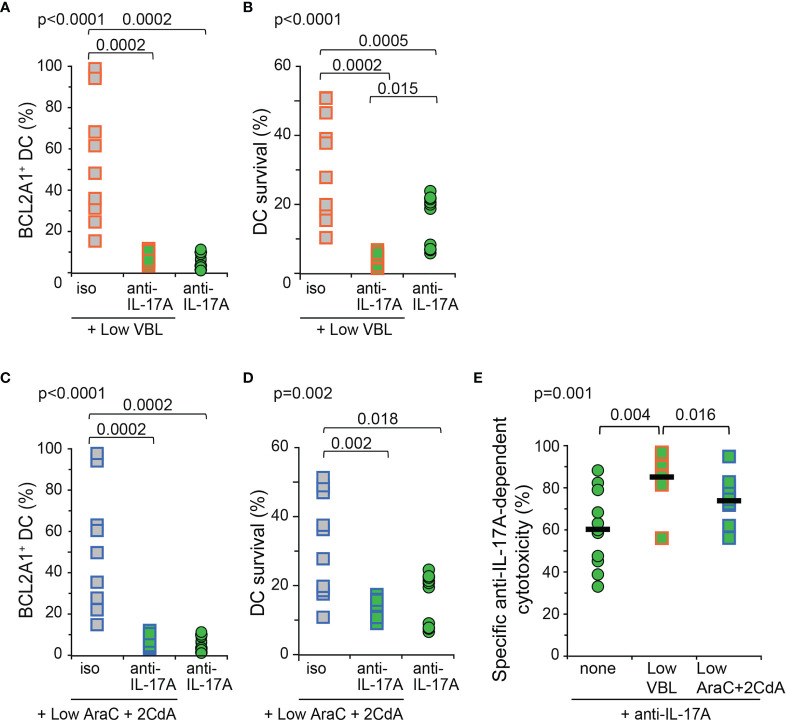
BCL2A1 expression and survival of Mo-DCs from LCH patients after combined *in vitro* treatment with toxic compounds and neutralizing anti-IL-17A antibodies. Mo-DCs from LCH patients were cultured with low doses of toxic compounds in the presence of isotype control (iso) or anti-IL-17A Abs (anti-IL-17A), and compared to Mo-DCs from LCH patients cultured with neutralizing IL-17A Abs alone. **(A, C)** Intracellular BCL2A1 staining was performed 12 hours before **(B, D)** DiOC_6_/PI staining, operated at day 7 and calculated per million cultured Mo-DCs. **(A–D)** Mo-DCs from LCH patients were incubated with low doses of **(A, B)** VBL or **(C, D)** AraC and 2CdA. **(E)** Specific anti-IL-17A-dependent cytotoxicity was calculated using [survival without anti-IL-17A – survival with anti-IL-17A]/survival without anti-IL17A x 100 for each 11 patients from the survival data shown in B and D. Bars represent the mean for all 11 patients. **(A–E)** The mean of triplicate values was plotted for each patient. SD were below 2%. One symbol corresponds to one patient. Mo-DCs from 11 LCH patients (p1b, p2b, p7c, p6b, p9, p12, p13, p14, p16, p19, p20) were analysed. Statistical analyses: the Kruskal-Wallis test with Steel-Dwass-Critchlow-Fligner post-test were used to compare the groups and calculate the p values.

Low doses of VBL or AraC and 2CdA toxic compounds had no detectable effect on LCH-DC survival (**Figure 3**). In the present preclinical *in vitro* study conducted with human primary Mo-DCs from LCH patients, we finally investigated the potential benefit of adding neutralizing anti-IL-17A antibodies to low doses of LCH-directed chemotherapeutic agents, in the attempt to mimic future therapies. Mo-DCs from LCH patients were cultured with low doses of toxic compounds in the presence of either isotype control or neutralizing anti-IL-17A antibodies, and then compared to DCs treated with anti-IL-17A antibodies. All conditions were analysed by flow cytometry for the intracellular expression of BCL2A1 and cell survival (**Figure 5**). The combination of anti-IL-17A neutralizing antibodies with low dose of VBL completely inhibited the expression of BCL2A1 (**Figure 5A**) and abrogated DC survival (<7%) of Mo-DCs from LCH patients (**Figure 5B**). This result was conclusive in all patients tested, including the patient (p7) whose Mo-DCs were resistant to high doses of VBL (**Figure 3C**). Therefore, neutralization of IL-17A overcame the resistance of this patient’s cells to VBL. The combination of anti-IL-17A with VBL was more efficient than anti-IL-17A alone (**Figure 5B**). In comparison, similar results were obtained after IL-17A blockade and the addition of low doses of the toxic compounds AraC and 2CdA (**Figures 5C, D**). The combination both decreased BCL2A1 expression and DC survival (**Figure 5D**). Lastly, we calculated the specific anti-IL-17A-dependent cytotoxicity for each patient (**Figure 5E**). To prevent DC survival from LCH patients, neutralisation of IL-17A was more efficient when combined to VBL than to AraC and 2CdA.

In conclusion, in this preclinical *in vitro* study performed on human Mo-DCs from LCH patients, blockade of IL-17A shows a beneficial effect when combined to a low, sub-clinical dose of VBL.

## Discussion

Pathogenic mechanisms and clinical manifestations of cancer, in particular of haemoproliferative disorders, may result from both the rate of proliferation and the ability of cancer cells to survive, even in the presence of chemotherapeutic agents. Different mechanisms may account for survival of pathogenic DCs and chemoresistance in LCH. Among them, pro-inflammatory IL-17A could play a central role. In this study, we show that DCs and MGCs in LCH lesions, as well as Mo-DCs from LCH patients, abnormally express BCL2A1/BFL1, a pro-survival member of the BCL2 family. We demonstrate that endogenous IL-17A stimulates BCL2A1 expression and long-term survival of Mo-DCs from LCH patients, *in vitro*. BCL2A1 expression was correlated with both IL-17A expression and LCH-DC survival. Novel therapeutic approaches are warranted in LCH, with the aims of improving survival as well as reducing the number of reactivations and sequelae associated with this disease, especially neurodegeneration (33). Here, we show for the first time that *in vitro* IL-17A blockade decreases both BCL2A1 expression and LCH-DC survival. Moreover, the combination of anti-IL-17A antibodies with low doses of chemotoxic drugs completely restores *in vitro* apoptosis of LCH-DCs. These preclinical findings may provide the rationale for novel therapeutic approaches, targeting IL-17A and related survival pathways, to be considered for future clinical trials.

Our results demonstrate that BCL2A1 staining improved delineation of LCH granulomas compared to CD1a staining. Indeed, staining for BCL2A1 expression provided a molecular marker to delineate the structure of LCH lesions, paving the way for future molecular single-cell studies. With the development of the new field called immunometabolism and knowing that redox status regulates the structure and the function of BCL2A1 (50), it would be interesting to study whether redox metabolism is a player between IL-17A and BCL2A1, empowering further the tissue lesions observed in LCH. Of note, BCL2A1 is not only expressed in pathogenic DCs of LCH lesions, but also in Mo-DCs from LCH patients.

We previously developed an *in vitro* model in which primary Mo-DCs from the peripheral blood of LCH patients are well characterized (8). By using this model, we proposed that (*i*) LCH is a Mo-DC-related disease rather than an LC-related disease, (*ii*) IL-17A is a source of inflammation in LCH lesions and (*iii*) LCH-DCs acquire the ability to survive and thus to accumulate. To our knowledge, we provide the first evidence that the extended survival of LCH-DCs is sustained by IL-17A-driven BCL2A1 expression. In addition to its pro-survival function and role in leukocyte development, BCL2A1 plays a pivotal role during immune response and inflammation (40). At steady-state, BCL2A1 is not expressed in myeloid cells, but it can be induced during myeloid differentiation and activation. Among the mechanisms of BCL2A1 induction, inflammatory stimuli, including pro-inflammatory cytokines also found in LCH, such as GM-CSF, IL-1β, TNF-α, IFN-γ and IL-17A, activate BCL2A1 gene expression and extend survival of macrophages and/or DCs (23, 35, 36). BCL2A1 is the main protein of the BCL2 family which is regulated by NF-κB (40). In Mo-DCs from healthy donors, downstream of the IL-17A receptor, NF-κB induces BCL2A1 expression (35). This mechanism induced in DCs from healthy donors by paracrine IL-17A may be activated by both paracrine and autocrine IL-17A in Mo-DCs cultured from LCH patients. Recent data indicate that downstream of the IL-17A receptor, the adaptor molecule ACT1 targets specific mRNA secondary structures of inflammatory genes induced by either IL-17A or other pro-inflammatory cytokines, such as IL-1β and TNF-α (51, 52). This original mechanism also targets IL-17A mRNA itself and is thought to be the major mechanism explaining the particularity of IL-17A i.e. its ability to sustain long-term chronic inflammation by stabilizing these mRNAs (52). It would be interesting to study whether some members of the BCL2 family, and especially BCL2A1, may offer the secondary structure targeted by ACT1 in their mRNA.

In addition to BCL2A1, Mcl-1 and BCL2 were more frequently overexpressed in haematological malignancies than Bcl-x_L_ (BCL2L1) and Bcl-w (BCL2L2), other anti-apoptotic members of the BCL2 family (37). Mcl-1 is constitutively expressed in Mo-DCs from both healthy donors (35, 36) and LCH patients, as demonstrated in this study. Immunohistological studies revealed that BCL2 is not expressed in normal skin. In LCH, BCL2 was first detected by immunohistochemistry and RNA *in situ* hybridization (53–56), but this data was not confirmed by transcriptomic analyses of CD207^+^ cells from LCH lesions (4). High expression of Bcl-x_L_ was detected in BRAFV600E-CD207^+^ cells from LCH lesions and decreased by BRAF or MEK inhibitors, which induce cell death (4, 11, 16). In Mo-DCs from LCH patients, we have documented the expression of BCL2A1, but neither BCL-2 nor BCL-x_L_ mRNAs, which are undetectable (data not shown). Expression of pro-survival BCL-2 members depends on cell type, location, differentiation stage and microenvironment, especially for BCL2A1 (39, 40). In addition to IL-17A, MAPK activating mutations could also support BCL2A1 expression in LCH lesions. Thus, a comprehensive molecular and immunohistological study in human LCH biopsies of such mutations, markers of LCH myeloid cells (CD207, CD1a, CD14, CD68) and members of the BCL-2 family, whether regulated by MAPK activating mutations or IL-17A, would provide important information to understand the heterogeneity of patient phenotype and ultimately how to conduct personalized treatment for a better care.

Although LCH often has a favourable outcome, some patients with disseminated disease still have an unacceptably high risk of death from LCH-driven multi-organ failure. VBL is successfully used as the standard first-line therapy for severe LCH (43, 44, 49). The combination of AraC and 2CdA is indicated as a second-line therapy for non-responsive patients or patients who escape VBL-mediated killing after completion of 6 or even 12 months of standard front line therapy (14, 16), and more recently MAPK inhibitors were also used as salvage therapy (46). BCL2A1 and Mcl-1 play a pivotal role in chemoresistance in a large number of haematological malignancies [see reviews (37, 39, 57)]. Our preclinical *in vitro* data demonstrated that VBL or AraC and 2CdA target Mcl-1 but not BCL2A1. As in other haematological malignancies, targeting one BCL2 member may lead to chemoresistance by fostering the upregulation of another pro-survival member (37). Although 14/20 samples arose from treated patients in the present study, we also investigated pre/un-treated LCH patients, and found high BCL2A1 levels in some of them. Therefore, LCH-DCs may escape the immune system and/or treatment to survive by regulating the expression of BCL2 members, especially BCL2A1. To substantiate this hypothesis, further investigations will be required on a larger cohort of treated versus untreated patients, and we propose to monitor BCL2 evolution according to disease progression.

We show here that neutralizing anti-IL-17A antibodies inhibits BCL2A1 expression and reduces DC survival although less efficiently than high doses of VBL or AraC and 2CdA. A combination of anti-IL-17A and chemotherapeutic agents decreased both BCL2A1 and Mcl-1 expressions and was highly effective at killing LCH-DCs, notably with suboptimal doses of VBL, thus demonstrating that targeting IL-17A offers a therapeutic advantage. Lowering the dose of VBL by adding anti-IL-17A biotherapy might represent an appealing therapeutic opportunity to limit the first-line treatment-related toxicity in LCH. It may be particularly interesting also in adult patients with LCH, in whom the use of VBL has been considered controversial, due to more frequent association with neuro-toxicity in some studies (58), but not in others (59).

Another alternative could be to target both Mcl-1 and BCL2A1 activities using peptide inhibitors or small molecules such as selective BH3-mimetics. While many Mcl-1 inhibitors have been generated, few are under clinical development, and none have yet been approved for clinical use (60, 61). Concerning BCL2A1, very few peptide inhibitors or small molecules are in development, though some have been tested *in vitro* and *in vivo* in animal models; no clinical development was reported (62). To date, no BH3-mimetic selectively targets BCL2A1 [see reviews (57, 62–64)]. Among the most studied BH3-mimetics, ABT-737 or its derivative ABT-263 mainly target BCL2, Bcl-x_L_ and Bcl-w, but neither BCL2A1 nor Mcl-1 (65). A recent study shows that ABT-263 is able to eliminate senescent cells strongly expressing BCL-2 and BCL-x_L_ proteins in a mouse multipotent hematopoietic progenitor cell model where LCH lesions contain the BRAFV600E mutation (66). Though initially effective in haematological malignancies, ABT-737 treatment has led to resistance owing to the alternative upregulation of BCL2A1 and Mcl-1 (67). In addition, pan-BCL2 and multiple-BCL2 inhibitors often have a very low clinical activity and/or high toxicity, thus necessitating the use of high selective inhibitors for each of the BCL2 members. However, dual or multiple combinations of individual inhibitors do not seem to be under clinical evaluation for haematological cancers (61).

Upregulation of LCH-DC survival pathways promotes an increase in pro-survival mutations, especially in the *BRAF, MAP2K1, ARAF and ERBB3* genes (15, 21, 25, 68). Although MAPK-inhibitors offer a promising option for treating LCH, these inhibitors seem to be ineffective at reducing BRAFV600E+ LCH cell counts, unlike most chemotherapy drugs, and may require the combination with other therapies (45, 69, 70). To improve therapeutic strategies, neutralizing IL-17A could be useful in LCH patients with or without MAPK-inhibitors according to the presence or absence of MAPK activating mutations. Our preclinical data strongly suggest that BCL2A1 expression is under the control of IL-17A, thus highlighting IL-17A as an innovative target to be neutralized. Current anti-IL-17A biotherapies (e.g. Secukinumab or Ixekizumab) have shown their effectiveness to control some chronic inflammatory disorders, such as rheumatoid arthritis [reviewed in (71–73)].

In conclusion, we provide the proof-of-concept that neutralizing IL-17A may limit accumulation of aggressive inflammatory LCH-DCs by targeting pro-survival BCL2A1 expression in addition to VBL, which targets Mcl-1. These preclinical *in vitro* data support the design of a specific clinical trial based on anti-IL-17A and VBL combination, with the potential to improve disease control in life-threatening LCH.

## Data Availability Statement

The original contributions presented in the study are included in the article/**Supplementary Material**. Further inquiries can be directed to the corresponding author.

## Ethics Statement

The studies involving human participants were reviewed and approved by Azienda di Rilievo Nazionale ed Alta Specializzazione Civico - Ospedale dei Bambini at Palermo (Italy), and “Regionala etikprövningsnämnden Stockholm” and “The Regional Ethical Review Board in Stockholm” (Sweden). Written informed consent to participate in this study was provided by the participants’ legal guardian/next of kin.

## Author Contributions

MA, J-IH, and CD designed the study. SOA, MBI, AB, GS, and NB contributed to data acquisition, analysis and interpretation. SOA, DG, MA, and J-IH ensure clinical management of patients and their samples. MA, J-IH, HV, and CD wrote the manuscript. All authors contributed to the article and approved the submitted version.

## Funding

This work was supported by grants from (FR) CNRS, INSERM, Université de Lyon 1, Institut Universitaire de France, Agence Nationale de la Recherche 2009-12, Fondation de France, Institut National contre le Cancer, Canceropole Lyon Auvergne Rhone Alpes (CLARA), ARC1 “santé” Region Rhone-Alpes (2014–15), Ligue Contre le Cancer Ardèche Drome and Rhone (2016-17); (EU) the EMJMD LIVE (Erasmus+ Mundus Joint Master Degree Leading International Vaccinology Education, award 2018-1484), co-funded by the EACEA (Education, Audiovisual and Culture Executive Agency) of the European commission; (IT) Ministero Sanità, Progetto di Ricerca Finalizzata 2008: “Getting deeper in histiocytosis”, Regione Toscana, Progetto di Ricerca Malattie Rare 2008, Associazione Italiana Ricerca Istiocitosi (AIRI); (SE) the Swedish Children’s Cancer Foundation, the Swedish Research Council, Märta och Gunnar V Philipson’s Foundation, the Cancer and Allergy Foundation of Sweden, Karolinska Institutet (KID project) and the Stockholm County Council (ALF project); (US) Histiocytosis Association of America 2008.

## Conflict of Interest

The authors declare that the research was conducted in the absence of any commercial or financial relationships that could be construed as a potential conflict of interest.

## Publisher’s Note

All claims expressed in this article are solely those of the authors and do not necessarily represent those of their affiliated organizations, or those of the publisher, the editors and the reviewers. Any product that may be evaluated in this article, or claim that may be made by its manufacturer, is not guaranteed or endorsed by the publisher.

## Supplementary Material

Supplementary Figure 1
*BCL2A1* mRNA relative expression in Mo-DCs (ctrl1, 2, 3, 5, 8) and in IL-17A-treated Mo-DCs [ctrl4, 6, as in (35)] from healthy donors, compared to *BCL2A1* mRNA relative expression in IL-17A-producing Mo-DCs from patients with LCH (p1a, p2a, p3, p5, p7a, p10). The dotted line indicates the significant expression threshold. Microarray analysis was performed using a high-density oligonucleotide array (Genechip human genome U133 Plus 2.0, Affymetrix). Labeled target for microarray hybridization was prepared using the Genechip expression 3’ Amplification One-cycle target labeling (Affymetrix). Briefly, total RNA (2 µg) was converted into double stranded cDNA with a modified oligo(dT)24-T7 promoter primer. After purification, cDNA was converted into cRNA and biotinylated using the IVT labeling kit (Affymetrix). Reaction was carried out for 16 hours at 37°C then at the end of incubation biotin-labeled cRNA was purified by the Genechip sample clean up module (Affymetrix). cRNA quantification was performed with a nanodrop and quality checked with the bioanalyzer 2100 (Agilent technologies). Hybridization was then performed following Affymetrix protocol (http://www.affymetrix.com). Briefly, 20 µg of labeled cRNA was fragmented, mixed in hybridization buffer (50 pM control oligo B2, 1X eukaryotic hybridization controls, 0,1mg/mL Herring sperm DNA, 0.5 mg/mL BSA and 1x hybridization buffer, 10% DMSO for a total volume of 300 µL), denaturated during 5 min at 95°C and hybridized on chip during 16 hours at 45°C with constant mixing by rotation at 60 rpm in an Genechip hybridization oven 640 (Affymetrix). After hybridization, arrays were washed and stained with streptavidin-phycoerythrin (Invitrogen Corporation) in a fluidic 450 (Affymetrix) according to the manufacturer’s instruction. The arrays were read with a confocal laser (Genechip scanner 3000, Affymetrix) and analyzed with GCOS software. Absolute expression transcript levels were normalized for each chip by globally scaling all probe sets to a target signal intensity of 500. The detection metric (presence, absence or marginal) for a particular gene was determined by means of default parameters in the GCOS v1.4 software (Affymetrix). Quality of RNA amplification and labeling were checked by using *B. subtilis* polyadenylated RNA spikes-in controls (Lys, phe, thr, dap) mixed to RNA sample before performing reverse transcription. Hybridization quality was checked by using *E. coli* biotinylated target (Bio B, BioC, BioD and CRE). Filtering of results was performed using Genespring v7.0 software (Agilent technologies Inc). Heat map was creating using Java TreeView v1.1.6r2.

Supplementary Figure 2 Representative flow cytometry analyses of BCL2A1 intracellular staining in Mo-DCs from LCH patients after incubation with either medium alone (none) or high doses of VBL (High VBL) or AraC and 2CdA (High AraC + 2CdA). Dot plots present SSC/FSC morphology to show the cells gated in G1 (left). Then, gated in G1 (right), dot plots present the intracellular staining with isotype antibody control (control), shown for medium alone (none) and also representative of the two other culture conditions (not shown). Finally, three dot plots present the intracellular staining with anti-BCL2A1 antibodies for the three culture conditions, as indicated above.

## Abbreviations

LCH, Langerhans Cell Histiocytosis; DCs, dendritic cells; Mo-DCs, monocytes-derived DCs; MGCs, multinucleated giant cells; MAPK, mitogen-activated protein kinase; IL-17A, interleukin-17A; rhIL-17A, recombinant human IL-17A; BCL2A1, BCL2-related protein A1; Mcl-1, myeloid cell leukaemia 1; VBL, vinblastine; AraC, Cytarabine; 2CdA: Cladribine.
