# Supplementary figures and images for: Neutralizing Anti-IL-17A Antibody Demonstrates Preclinical Activity Enhanced by Vinblastine in Langerhans Cell Histiocytosis
Source: Front Oncol. 2022 Jan 21;11:780191. doi: 10.3389/fonc.2021.780191 (PMC8814633; doi:10.3389/fonc.2021.780191)

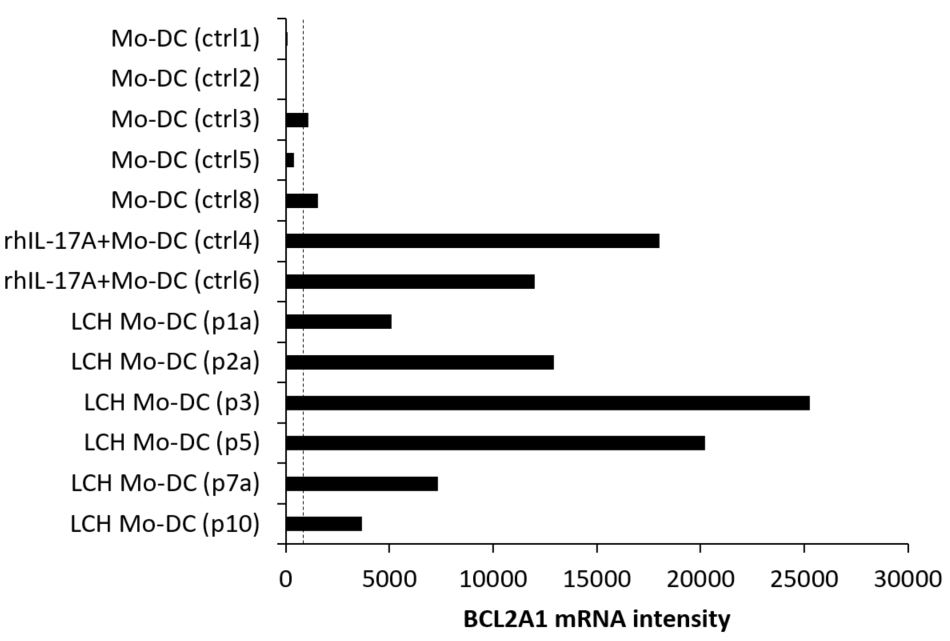

Supplement: Supplementary Figure 1 — BCL2A1 mRNA relative expression in Mo-DCs (ctrl1, 2, 3, 5, 8) and in IL-17A-treated Mo-DCs [ctrl4, 6, as in (35)] from healthy donors, compared to BCL2A1 mRNA relative expression in IL-17A-producing Mo-DCs from patients with LCH (p1a, p2a, p3, p5, p7a, p10). The dotted line indicates the significant expression threshold. Microarray analysis was performed using a high-density oligonucleotide array (Genechip human genome U133 Plus 2.0, Affymetrix). Labeled target for microarray hybridization was prepared using the Genechip expression 3’ Amplification One-cycle target labeling (Affymetrix). Briefly, total RNA (2 µg) was converted into double stranded cDNA with a modified oligo(dT)24-T7 promoter primer. After purification, cDNA was converted into cRNA and biotinylated using the IVT labeling kit (Affymetrix). Reaction was carried out for 16 hours at 37°C then at the end of incubation biotin-labeled cRNA was purified by the Genechip sample clean up module (Affymetrix). cRNA quantification was performed with a nanodrop and quality checked with the bioanalyzer 2100 (Agilent technologies). Hybridization was then performed following Affymetrix protocol (http://www.affymetrix.com). Briefly, 20 µg of labeled cRNA was fragmented, mixed in hybridization buffer (50 pM control oligo B2, 1X eukaryotic hybridization controls, 0,1mg/mL Herring sperm DNA, 0.5 mg/mL BSA and 1x hybridization buffer, 10% DMSO for a total volume of 300 µL), denaturated during 5 min at 95°C and hybridized on chip during 16 hours at 45°C with constant mixing by rotation at 60 rpm in an Genechip hybridization oven 640 (Affymetrix). After hybridization, arrays were washed and stained with streptavidin-phycoerythrin (Invitrogen Corporation) in a fluidic 450 (Affymetrix) according to the manufacturer’s instruction. The arrays were read with a confocal laser (Genechip scanner 3000, Affymetrix) and analyzed with GCOS software. Absolute expression transcript levels were normalized for each chip by globally scaling [file Image_1.jpeg]

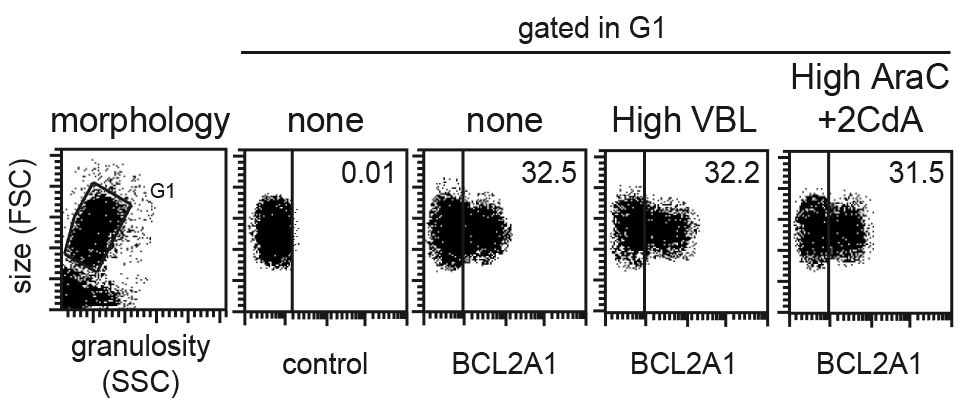

Supplement: Supplementary Figure 2 — Representative flow cytometry analyses of BCL2A1 intracellular staining in Mo-DCs from LCH patients after incubation with either medium alone (none) or high doses of VBL (High VBL) or AraC and 2CdA (High AraC + 2CdA). Dot plots present SSC/FSC morphology to show the cells gated in G1 (left). Then, gated in G1 (right), dot plots present the intracellular staining with isotype antibody control (control), shown for medium alone (none) and also representative of the two other culture conditions (not shown). Finally, three dot plots present the intracellular staining with anti-BCL2A1 antibodies for the three culture conditions, as indicated above. [file Image_2.jpeg]
